# Case Report: Malignant phyllodes tumor of the breast with heterologous osteosarcomatous differentiation and literature review

**DOI:** 10.3389/fonc.2025.1635114

**Published:** 2025-09-23

**Authors:** Liang Guo, Jing Wang, Xiang Li, Xin Dong, Xiaoqian Lu, Dianbo Cao

**Affiliations:** ^1^ Department of Pathology, The First Hospital of Jilin University, Changchun, China; ^2^ Department of Radiology, The First Hospital of Jilin University, Changchun, China

**Keywords:** breast, phyllodes tumor, osteosarcomatous differentiation, pathology, imaging

## Abstract

Phyllodes tumor (PT) of the breast is a rare fibroepithelial tumor characterized by the proliferation of both epithelial and stromal components. The presence of osteosarcomatous differentiation within the sarcomatous stroma is exceptionally uncommon and typically portends a poor prognosis. However, the biological behavior of malignant phyllodes tumors (MPT) exhibiting heterologous osteosarcomatous differentiation requires further investigation. A 59-year-old woman presented with a one-month history of a left breast lump. Mammography, chest CT, ultrasonography and MRI identified a mass measuring 3.7cm×5.6cm×4.7cm. Notably, the time-intensity curve derived from DCE-MRI demonstrated a pattern of rapid initial enhancement followed by slow washout. The patient underwent wide local excision, and postoperative histopathology examination confirmed MPT with heterologous osteosarcomatous differentiation, predominantly composed of neoplastic bone. The patient declined adjuvant therapy and was managed with regular follow-up. Twelve months later, she returned with a recurrent breast mass. Mammography and chest CT showed a calcified mass measuring 6.3cm×6.5cm, resembling the previous lesion. A total mastectomy accompanied by partial resection of the pectoral major muscle was performed. The histopathological examination of the second specimen was consistent with the initial diagnosis. Following the second surgery, the patient received four cycles of chemotherapy and was maintained on regular surveillance. Ten months later, follow-up CT imaging revealed extensive pleural effusion with complete passive atelectasis of the left lung, along with scattered patchy and curvilinear calcifications along the mediastinal and parietal pleura. The patient declined further chemotherapy and opted for traditional Chinese medicine, and she died three months later. MPT of the breast with heterologous osteosarcomatous differentiation is an exceedingly rare entity with a poor prognosis despite aggressive therapeutic interventions. Different from previously reported cases, our case elucidates the tumor’s biological behavior through serial image follow-up, and highlights its hypervascularity which was not detected by color Doppler ultrasound but was clearly demonstrated on DCE-MRI.

## Introduction

Phyllodes tumor (PT) of the breast is a rare fibroepithelial neoplasm marked by the proliferation of both epithelial and stromal components. Originating from the intralobular and periductal stroma, PT pathogenesis involves complex epithelial-stromal interactions. Molecular analyses of both components have revealed coordinated alterations in signaling networks that drive tumorigenesis. Key mutations within the stromal compartment propel its overgrowth and malignant progression, definitively distinguishing PT from fibroadenoma (1, 2). Accounting for only 0.3%-1.0% of all primary breast tumors, PT exhibits a higher incidence among Asian populations compared to Western cohorts (3). Based on histopathological features, PT is categorized into benign, borderline, and malignant with corresponding recurrence rates of 10%-17%, 14%-25%, and 23%-30%, respectively (4). PT typically manifests as a unilateral, large (often >10cm), circumscribed breast mass. While sonography, mammography, CT, and MRI may reveal a lobulated mass, occasionally with high calcified attenuation, but these imaging characteristics contribute limited value in predicting tumor grade. Diagnosis and grading rely mainly on histopathological evaluation. As reported in the literature, 10%-15% of phyllodes tumors are malignant (5). According to the World Health Organization(WHO) Classification of Breast Tumors (2019), diagnostic criteria for malignant phyllodes tumor (MPT) include marked stromal nuclear pleomorphism, stromal overgrowth, high mitoses (≥5 mitoses/mm^2^), increased stromal cellularity, and an infiltrative border. In even rarer instances, sarcomatous stromal elements within PT may encompass angiosarcoma, chondrosarcoma, leiomyosarcoma, osteosarcoma, and rhabdomyosarcoma, frequently indicative of an adverse clinical outcome. Herein, we report a case of breast MPT comprised predominantly of heterologous osteosarcoma, systemically reviewing its clinical presentation, imaging features, pathological characteristics, and disease course. Additionally, we performed a comprehensive literature review via PubMed for scattered case reports of breast PT with osteosarcomatous differentiation published since 2000. By analyzing these collective data, we aim to advance the understanding of this disease entity to facilitate accurate diagnosis and optimal management.

## Case presentation

**Figure 1:**
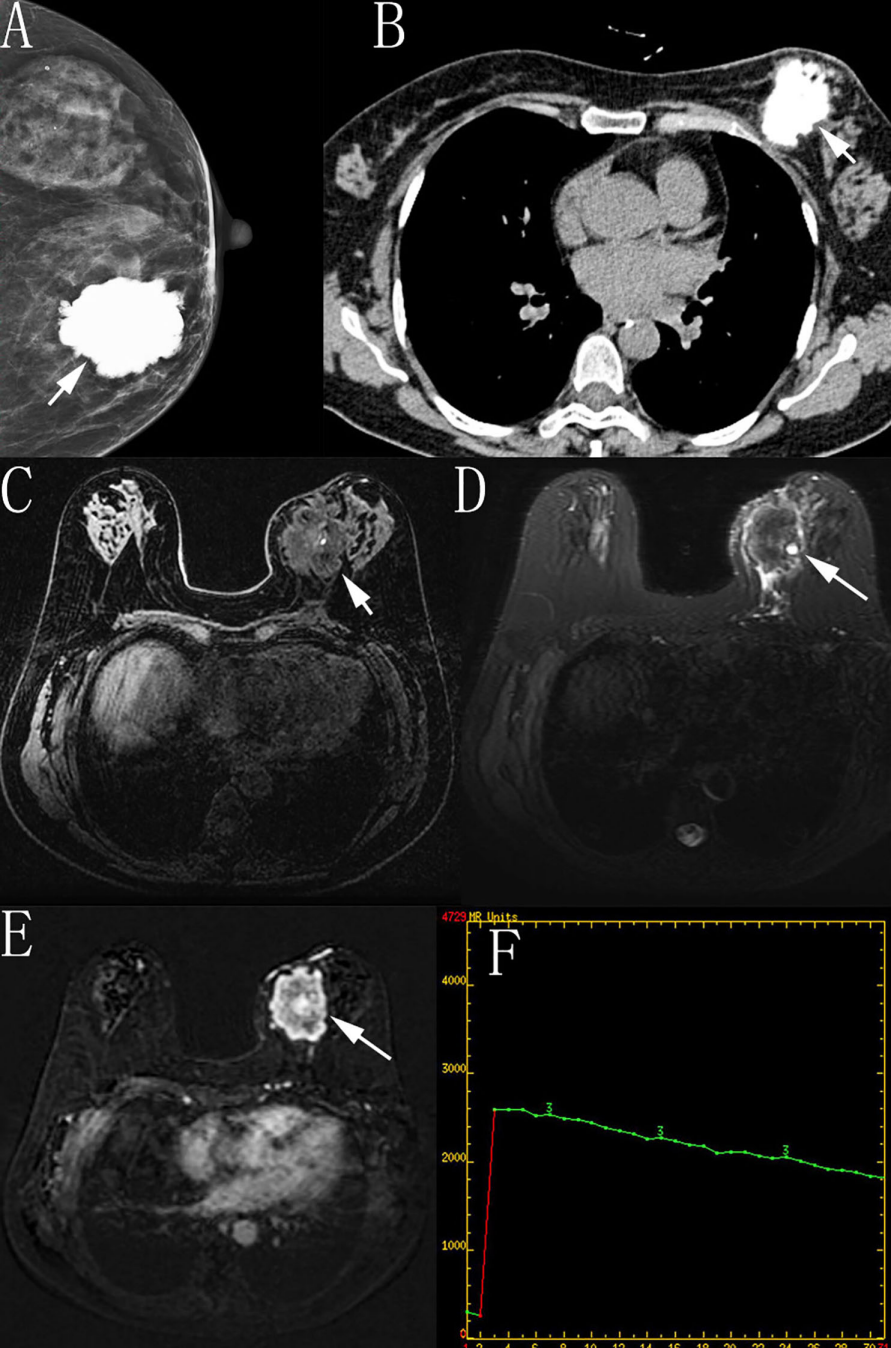
**(A-F)** Mammography and chest CT showed a hyperdense mass with lobulated borders **(A, B)**. On fat-suppressed T1-weighted image, the tumor was hypointense or isointense, while on fat-suppressed T2-weighted image it was mildly hyperintense and hypointense along with circular hyperintensity **(C, D)**. Contrast-enhanced MRI showed uneven enhancement, and the time-intensity curve on DCE-MRI was of rapid uptake and slow reduction type **(E, F)**.

A 59-year-old female complaining of a left breast lump for one month was admitted to the Department of Breast Surgery on December 14, 2020. She had been postmenopausal for 12 years and denied any history of hormone therapy. She first noticed a nodule in her left breast 1 month prior which had progressively enlarged over the preceding 3 weeks. Physical examination revealed a 5.0cm×4.0cm firm, movable mass in the lower inner quadrant of the left breast, with no associated skin changes. The axillary lymph nodes and contralateral breast were normal. Mammography showed a hyperdense mass with lobulated borders (Breast Imaging Reporting and Data System BI-RADS 3, indicating probably benign) (**Figure 1A**). Ultrasonography revealed a solid mass without obvious internal vascularity on color Doppler (BI-RADS 4, indicating suspicious for malignancy). Chest CT (**Figure 1B**) showed a lobulated hyperdense mass with diffuse calcification, and the left thorax was normal, with no evidence of chest wall invasion aside from displacement of the adjacent pectoralis major muscle. MRI demonstrated a well-defined and lobulated lump measuring 3.7cm×5.6cm×4.7cm. The tumor exhibited iso- or hypointense signal intensity to normal breast tissue on fat-suppressed T1-weighted images (**Figure 1C**). Fat-suppressed T2-weighted images showed areas of mild hyperintensity and hypointensity, surrounded by a rim of high signal intensity (**Figure 1D**). Dynamic contrast-enhanced MRI(DCE-MRI) revealed heterogeneous enhancement (**Figure 1E**), with a time-intensity curve indicative of rapid enhancement and slow washout (BI-RADS MR 5, highly suggestive of malignancy) (**Figure 1F**). Cerebral and abdominal CT scans showed no evidence of distant metastasis. Given the extensive calcification observed on mammography and CT, obtaining an adequate specimen for pathological diagnosis via needle biopsy was challenging. Due to the patient’s concern over potential tumor dissemination during biopsy, she declined this procedure. Consequently, excisional biopsy was pursued as an alternative for definitive diagnosis.

**Figure 2:**
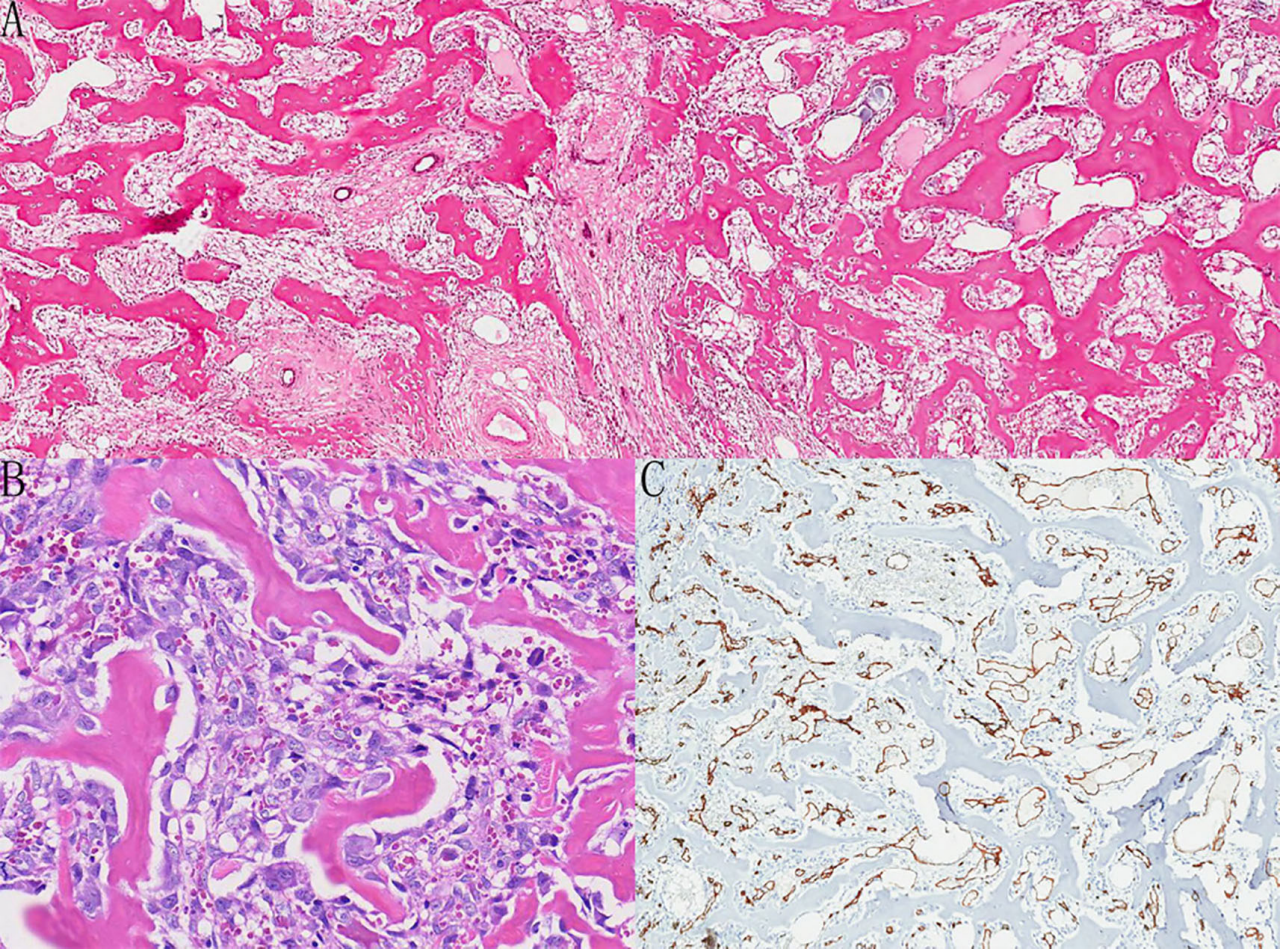
**(A-C)** The woven bone with a lace-like pattern was surrounded by sarcomatoid cells and benign glands [**(A)** HE×40 **(B)** HE×200]. The CD34 immunohistochemistry highlighted the abundant vasculatures among the trabeculae of the woven bone [**(C)** IHC×40].

The patient underwent wide local excision on December 17, 2020. Intraoperatively, the mass was found not to involve the thoracic wall muscles. The resected tumor measured 5.5cm×4.0cm×3.5cm and exhibited a bony gross appearance. Microscopically, the osteogenic tumor consisted primarily of well-differentiated bone trabeculae fusing with abundant microvasculatures and interspersed benign glandular structures (**Figures 2A, B**). Adjacent to the trabeculae, sarcomatoid cells displayed significant atypia and hypercellularity. The tumor border was circumscribed and infiltrative. Immunohistochemical analysis showed that tumor cells were negative for pan-cytokeratin (CKpan), while benign ductal epithelium was focally positive. Tumor cells expressed SATB2 and SMA but were negative for P63. The Ki-67 reached 40% in the hotspot area. CD34 staining presented microvascular plexus within the bone trabeculae (**Figure 2C**). After thorough examination of the tumor bed, a benign epithelial component was identified, confirming the final diagnosis of MPT with heterologous osteosarcomatous differentiation. The patient declined adjuvant chemotherapy and radiotherapy at that time.

**Figure 3:**
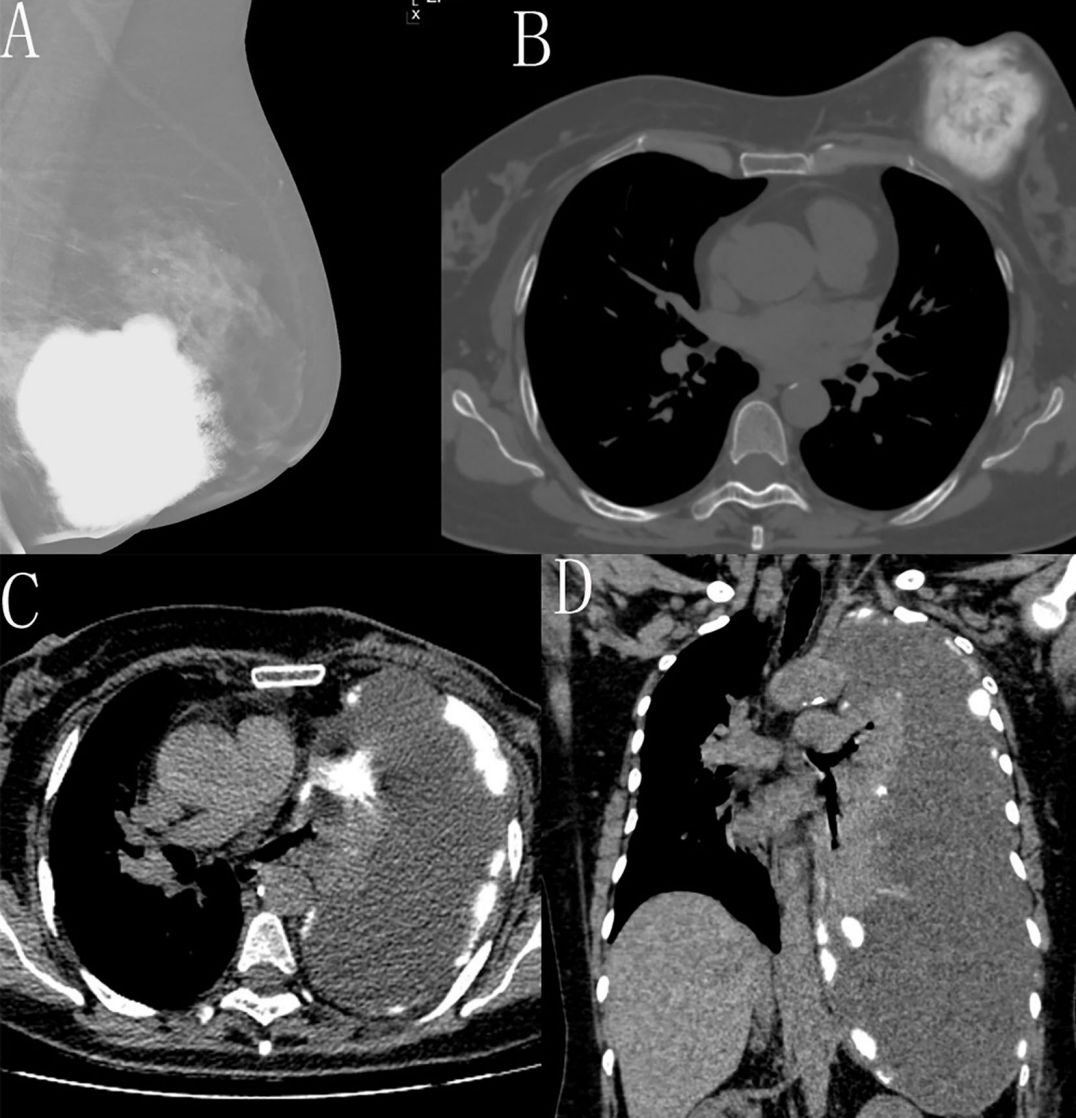
**(A-D)** Mammography and chest CT one year later showed a 6.3×6.5cm calcified mass **(A, B)**. Axial and coronal CT on 23 months after the first operation showed the atelectasis of the left lung associated with a large amount of pleural effusion and scattered pleural calcifications **(C, D)**.

On November 19, 2021, the patient returned with a recurrent mass in the left breast. Four months ago, she noticed a nodule in the left breast, then the nodule progressively enlarged. Physical examination revealed an 8.0cm×5.0cm firm mass with ill-defined borders. The axillary lymph nodes and contralateral breast remained unremarkable. Mammography showed a 6.3cm×6.5cm calcified mass (BI-RADS 3) (**Figure 3A**). There was no evidence of distant metastasis on cerebral, thoracic (**Figure 3B**), and abdominal CT. Based on clinical presentation and previous history, a diagnosis of tumor recurrence was made, and radical surgical intervention was recommended following multidisciplinary discussion. On November 25, 2021, the patient underwent total mastectomy and partial excision of the pectoralis major muscle. Gross appearance showed an 8.0cm×8.0cm×7.5cm bone-like tumor situated beneath the nipple adjacent to the superficial fascia. The microscopic morphology of the recurrent tumor resembled that of the initial specimen. Pathological diagnosis confirmed recurrent MPT with heterologous osteosarcomatous differentiation. Postoperatively, the patient received four cycles of chemotherapy and was regularly monitored.

On October 15, 2022, surveillance CT showed a large amount of pleural effusion with complete passive atelectasis of the left lung, accompanied by patchy and curvilinear calcifications along the mediastinal and parietal pleura (**Figures 3C, D**), suggestive of extensive pleural metastases. Given the clinical and imaging findings, a diagnosis of distant metastasis was made, and she commenced traditional medicine therapy.

**Figure 4:**
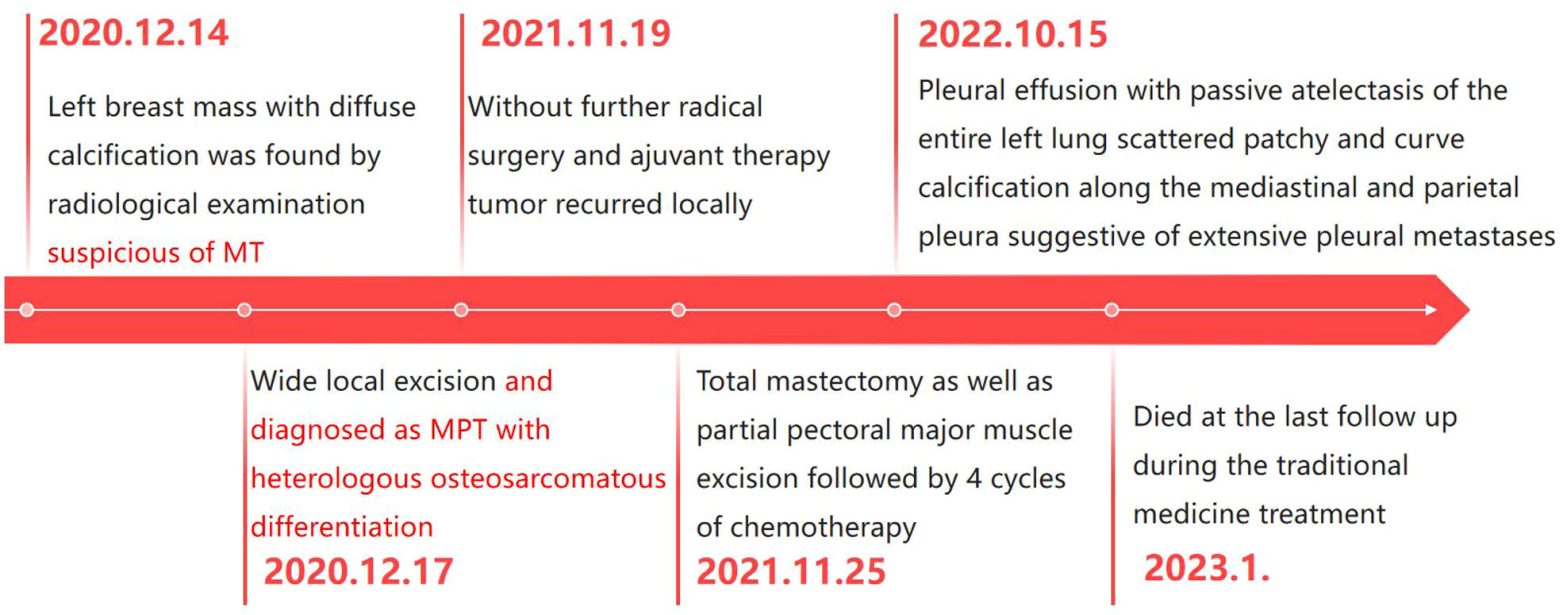
The timeline summarizing the main events of this case report.

In January 2023, the patient was deceased at the last follow-up. **Figure 4** outlines the timeline of the major events in this case.

## Discussion

As a rare entity, heterologous sarcomatous differentiation of MPT encompasses liposarcoma (excluding well-differentiated subtypes), osteosarcoma, chondrosarcoma, fibrosarcoma, or rhabdomyosarcoma component. A survey of 213 pathologists from 29 countries indicated that only 170 had encountered heterologous elements in MPT during their practice, with the incidence as follows: liposarcoma (53/170, 31.2%), chondrosarcoma (49/170, 28.8%), osteosarcoma (31/170, 18.2%), and rhabdomyosarcoma (17/170, 10.0%) (6). In English literature, most reports are isolated case with limited data. The clinical presentation and course of patients vary depending on the type of heterologous element. In 1999, Silver & Tavasolli reviewed the clinicopathological features and outcomes of 22 cases of MPT with osteosarcomatous differentiation (7). They reported a mean patient age of 60y (range: 40y-83y) and a mean tumor size of 6.4cm (range:1.9cm-19cm). Half of the tumors were grossly circumscribed and lobulated. Histologically, osteosarcomatous elements were categorized as fibroblastic (50%, featuring spindled malignant cells arranged in a storiform pattern), osteoclastic (27%, abundant non-neoplastic giant cells), or osteoblastic (23%, predominantly neoplastic bone matrix). Heterologous components constituted 25% to 100% of the tumor area. Notably, 43% of the reported patients experienced recurrence or metastasis and died within 12 months. Their analysis revealed that tumor size greater than 5cm and/or histological subtype of osteoclastic/osteoblastic were closely related to poor prognosis.

Since 2000, including the present case, 27 cases have been reported in the English literature (**Table 1**). In summary, the mean patient age was 53.2y (range:24y-76y). Tumors occurred in the left breast in 14 cases (52%) and the right breast in 13 (48%). The mean tumor size was 8.8cm (range:3cm-22.8cm, from 26 surgical cases, one autopsy case without documented tumor size).Ten patients (37%) died from tumor recurrence within 2.5 to 40 months post-surgery, among these, one patient died of recurrence without recorded time after initial surgery. Two patients (7%) were alive with recurrent tumor at last follow-up (5 and 36 months, respectively), while 11 (41%) were disease-free at last follow-up (ranging from 6 to 85 months). Four patients (15%) were lost to follow-up.

**Table 1:** Case Summary of breast malignant phyllodes tumor with osteosarcomatous differentiation since 2000 (including present case).

Authors	Year	Age	Site	Size (cm)	Image manifestations	Treatment	Final pathology	Follow up
Jha et al. (8)	2023	32	L	10	US: irregularly shaped hypoechoic lesion with partially circumscribed and microlobulated margins	Total mastectomy	Osteosarcoma(30%) with an osteoblastic component originating from MPT (70%)	NA
Ko (9)	2023	52	R	7	Mammography: high-dense mass with coarse and amorphous macro- and microcalcifications US: heterogeneous solid and cystic mass MRI: irregular-shaped multi-cystic complex mass with a predominantly cystic appearance and peri-lesional edema	Modified radical mastectomy with axillary dissection without further therapy such as radiation or chemotherapy	MPT with heterologous osteosarcoma and chondrosarcomatous differentiation	8 months after surgery, stable
Ali et al. (10)	2023	51	R	14.9	US: a lobulated mass with heterogeneous enhancement, abutting the pectoralis muscle	Total mastectomy followed by radiotherapy	MPT with mixed osteosarcomatous and rhabdomyosarcomatous elements	6 months, no recurrence or metastasis
Bhandari et al. (11)	2023	55	R	NA	Autopsy record	Mastectomy	MPT showing foci of osteosarcomatous differentiation	No time or image documented, died from widespread metastasis of gastrointestinal tract
Laforga et al. (12)	2020	56	L	16	US: tumor occupying the entire breast	Radical mastectomy and subsequent chemotherapy	MPT with heterologous osteosarcomatous differentiation and aneurysmatic bone cyst-like features	31 months, died from pleural effusion and multiple lung metastases
Wu et al. (13)	2020	58	R	15	MRI: breast occupation CT: recurrence on the chest wall 2.5 months later	Expanded resection, radiotherapy and apatinib treatment	MPT with heterologous chondro- and osteosarcomatous dedifferentiation	2.5 months, multiple bone metastases on PET-CT. 6 months, died, suspicious of coexistent brain metastasis
Berkesoglu et al. (14)	2020	55	R	15	PET-CT: protruding lesion from the breast	Mastectomy and chemoradiotherapy	MPT with osteosarcomatous dedifferentiation	40 months, died from metastatic lesion
Patel et al. (15)	2019	45	R	22.8	US: lobulated mass of mixed echogenicity with areas of necrosis	Total mastectomy and chemoradiotherapy	MPT with osteosarcomatous dedifferentiation	Three years later, metastases to the skull, another 49 months after treatment, disease-free
Tokoyoda et al. (16)	2018	52	L	7	CT: a nodular calcified mass	Surgery and following postoperative radiotherapy	MPT with dominant osteosarcomatous differentiation	1 year, died from metastasis to the heart
Sarkar et al. (17)	2016	24	R	5	NA	Simple mastectomy with following radical mastectomy and chemotherapy	MPT with heterologous chondro- and osteosarcomatous dedifferentiation	Five months later, recurrence and dissemination of subcutaneous nodules, left mediastinal mass, and a necrotic axillary node on CT
Patil Okaly et al. (18)	2015	40	L	5	NA	Simple mastectomy	MPT with heterologous chondro- and osteosarcomatous dedifferentiation	1 year, disease-free
Warrier et al. (19)	2015	50	L	11	Mammogram and ultrasound: occupied lesion	Mastectomy and radiotherapy	MPT with heterologous chondro- and osteosarcomatous dedifferentiation and DCIS	2 years, disease-free
Warrier et al. (19)	2015	53	L	3.3	US: solid mass with a heterogeneous echo pattern, cystic spaces and well-defined margins.	Wide local excision	MPT with heterologous lipo- and osteosarcomatous dedifferentiation	2 years, disease-free
Mačák et al. (20)	2014	71	R	10	NA	Mastectomy	MPT with osteosarcomatous differentiation	3 years, died from right ventricular apex metastasis and circulatory failure
Phalak et al. (21)	2013	63	L	7	Mammogram: a lobulated high-dense mass with partially circumscribed, partially obscured margins and associated coarse heterogeneous calcifications US: a round hypoechoic mass with associated vascularity and multiple strong echogenicity	Wide excision following adjuvant chemotherapy	MPT with osteosarcomatous differentiation	10 months after the operation, pleural metastases
Singhal et al. (22)	2011	40	L	6	Mammogram: a well-defined mass with lobulated margins and areas of calcification similar to bone, fine eggshell calcification around the tumor	Simple mastectomy	MPT with heterologous chondro- and osteosarcomatous dedifferentiation	5 years, disease-free
Reisenbichler et al. (23)	2009	55	L	14.5	NA	Modified radical mastectomy	MPT with osteosarcomatous differentiation	6 months, disease-free
Tomas et al. (24)	2007	71	R	3.3	NA	Radical mastectomy and chemotherapy	MPT with osteo-, chondro- and liposarcomatous differentiation	1 year, disease-free
Ribeiro-Silva et al. (25)	2006	49	R	8	NA	Radical mastectomy	MP with osteosarcomatous differentiation	Two months after the mastectomy, recurrence. 8 months after surgery, metastasized to the liver, brain, lungs, and skin of the right forearm. 1 year later, died
Sando et al. (26)	2006	49	R	12	NA	Modified radical mastectomy	MPT with osteosarcomatous differentiation	Nine months after surgery died from multiple pulmonary metastases
Choudhary et al. (27)	2006	76	R	4	Mammogram: a well-defined lobulated mass with dense calcification. US: difficult to interpret vascularity because of the calcification producing significant acoustic shadowing	Total mastectomy	MPT with osteosarcomatous differentiation	Increased uptake around the right hip and medial compartment of both knees and a focal spot of intense uptake overlying the right anterior chest wall on the bone scan. NA
Bhartia et al. (28)	2005	45	L	3	Mammogram and US: a soft tissue mass with coarse macrocalcification	Simple mastectomy and subsequent chemoradiotherapy	MPT with osteosarcomatous differentiation	19 months, died from persistent right pleural effusion secondary to pleural metastases, nodules in the lung with increasing calcification on CT
Mukherjee et al. (29)	2004	51	R	6	Mammography: an asymmetrical density with scattered microcalcifications. US: a heterogenous mass with solid and cystic components and evidence of increased vascularity	Total mastectomy and chemotherapy	MPT with osteosarcomatous differentiation	NA
Tsubochi et al. (30)	2004	54	L	8	Mammography: soft mass without calcification	subcutaneous mastectomy	MPT with osteosarcomatous differentiation	One year after mastectomy, bilateral pulmonary tumors with calcification (metastasis), another 2 years after lung surgery, disease-free
Fischer et al. (31)	2003	66	L	3.9	Mammogram: a radiodense area with calcifications. US: a complex mass with irregular margins, with significant shadowing indicating the presence of calcium	Lumpectomy	MPT with osteosarcomatous differentiation	NA
Matsuo et al. (32)	2001	64	L	5	Mammography: an irregular tumorous lesion with coarse calcifications	surgery	MPT with osteosarcomatous differentiation	6 months, disease-free
Present case		59	L	5.5	Detailed data in this manuscript	Surgery and chemotherapy	MPT with osteosarcomatous differentiation	25 months, Died of metastasis

R, right; L, left; MPT, malignant phyllodes tumor; US, ultrasonography; CT, computed tomography; MRI, magnetic resonance imaging; DICS, ductal carcinoma in situ; NA, not available.

According to the cases listed in **Table 1**, imaging examinations revealed calcification in 10 tumors (9, 16, 21, 22, 27–29, 31, 32), and only 2 cases had vascular signals within the tumors (21, 29). Among recurrent tumors, two cases exhibited calcification on follow-up imaging (30, 32). Of the metastatic cases, pleural metastasis was observed in 3 patients (12, 21, 28), pulmonary metastasis in 2 (26, 30), cardiac metastasis in 2 (16, 20), bone and brain metastasis in 1 (13), skull metastasis in 1 (15), joint metastasis in 1 (27), and widespread metastasis in 3 (11, 17, 25); one case had metastasis with no documented location (14). Among the 9 reported cases with calcification on image, 2 died from the disease, 2 developed metastasis, 4 were alive with follow-up period shorted than 8 months, and only 1 remained disease-free at 5 years (9, 16, 21, 22, 27–29, 31, 32). The case with both calcification and internal vascularity spread to diffuse pleura within ten months after surgery (21). Including our present case, we speculate that the coexistence of calcification and increased vascularity may be a high-risk factor for recurrence, warranting more aggressive medical intervention. More young patients with larger tumors have been recorded in the literature since 2000. Advances in treatment may have contributed to improved overall survival and prolonged disease-free periods. However, the limited reported cases and lack of original data precluded a detailed statistical analysis. Further accumulation of well-documented cases is needed to identify clinically and pathologically significant prognostic factors.

Based on the WHO 2019 diagnostic criteria, the diagnosis of MPT is very strict. A definitive pathological diagnosis requires the presence of all characteristic morphological features, including marked stromal nuclear pleomorphism, stromal overgrowth, high mitoses (≥5 mitoses/mm^2^), increased stromal cellularity and an infiltrative border. However, the presence of malignant heterologous elements-such as liposarcoma (excluding well-differentiated liposarcoma), osteosarcoma, chondrosarcoma, fibrosarcoma, or rhabdomyosarcoma-allows for a diagnosis of MPT with heterologous differentiation, irrespective of whether the conventional histopathological criteria are fully met.

Microscopic patterns of our case including primary and recurrent neoplasm, are mainly composed of osteosarcomatous neoplastic bone. The foci of benign epithelial components confirmed the biphasic nature of the tumor. Abundant microvasculature was observed among bone trabeculae. Heterologous components constituted nearly 100% of the tumor tissue in both lesions. These pathological features help clarify the initially perplexing imaging findings: significant calcified attenuation on mammography and CT limited the acquisition of additional diagnostic information, even with contrast-enhanced CT. Conversely, the enhancement pattern on contrast-enhanced MRI provided valuable clues regarding malignancy. The bony matrix attenuated ultrasound wave, and the blood flow signals were unable to be detected on colored Doppler ultrasound. Our patient experienced disease progression despite aggressive treatments. This is in keeping with the dismal prognosis of the neoplastic bone-forming type of the heterologous element in the literature (7).

The main differential diagnosis for MPT is metaplastic carcinoma. The essential diagnostic criterion is the absence of a conventional infiltrating carcinoma component with mesenchymal differentiation. In our case, we did find small foci of CKpan-positive epithelial elements in the CKpan-negative osteosarcoma areas, but the epithelial element exhibited its benign feature with a low Ki-67 index and no obvious mitosis. So, the metaplastic carcinoma was ruled out. Other differentials include primary or metastatic osteosarcoma of the breast. The incidence of primary breast osteosarcoma varies greatly. According to the data from the Armed Force Institute of Pathology (AFIP), only 50 cases were reported between 1957 and 1995 (33). However, there was only 1 breast osteosarcoma in the database of Mayo Clinic from 1910 to 2000 (34, 35) and 3 cases in M.D Anderson Cancer Center from 1947 to 1990 (35). These discrepancies likely reflect evolving diagnostic criteria over time. According to WHO 2019 guidelines, a definitive diagnosis requires thorough sampling to exclude any epithelial component and to rule out metastatic tumor based on clinical history. Thus, meticulous identification of benign epithelial elements is vital to differentiate MPT with osteosarcomatous components from primary breast osteosarcoma.

The marked vascularization within the neoplastic bone is a hallmark of the present case. However, conventional ultrasound was unable to visualize these intratumoral vessels due to obscuration by the surrounding bony matrix. Contrast-enhanced MRI proved to be a more appropriate modality to identify the intrinsic nature of the tumor. Alternative diagnostic procedures such as DCE-MRI should be ordered when ultrasound findings are inconclusive. In breast carcinoma, time-intensity curves are characterized by rapid uptake and washout, often accompanied by focal calcifications. However, the tumor in our patient showed rapid uptake and slow washout with diffuse calcification. This point may be a meaningful diagnostic indicator and influence therapeutic decision. It has been reported that high microvessel density (MVD) correlates with a good response to chemotherapy in osteosarcoma (36). Moreover, novel treatment strategies such as nanoparticle-based targeting of tumor vasculature are under development (37). Therefore, accurate assessment of the vascular patterns within the tumor is important for oncologists to make an effective treatment plan.

Complete resection of the tumor remains the primary treatment for breast MPT. Following the initial extensive local excision, the patient experienced fatigue and expressed concern regarding potential side effects of chemotherapy and radiotherapy. The lack of adjuvant treatment post-surgery contributed to rapid recurrence and accelerated disease progression. Clinicians should ensure patients are fully aware of the aggressive characteristics of the tumor and take more radical measures to control the disease. The communication between the physician and the patient is crucial and an experienced psychologist may help the treatment plan proceed smoothly. Actually, after the first recurrence, the patient acknowledged the malignancy’s severity and consented to chemotherapy. Nonetheless, this did not hinder the progression of the disease. The recurrence of the disease is indicative of a poor prognosis. It has been shown that both chemotherapy and radiotherapy have limited efficacy against sarcomatous components. Provided the patient’s condition permits following comprehensive evaluation, more radical resection may still be of value. All reported cases in the literature underwent surgery to remove the primary tumor. After the surgery, 6 received chemotherapy, 3 received radiotherapy, 3 received concurrent chemoradiotherapy, and 1 received radiotherapy combined with targeted therapy. Although the treatment data are limited, total removal of the primary tumor with adjuvant chemoradiotherapy has been associated with improved overall survival. According to our literature review on MPT since 2000, rates of recurrence and metastasis have declined markedly, and the overall survival has significantly increased. Recent studies suggest that nanoparticles with high affinity for tumor vasculature may enhance ultrasonic signals and facilitate earlier detection. Novel treatment targeting the tumor microvasculature may reduce the chemo-resistance of the sarcoma and improve the overall survival. For advanced disease, such innovative strategies may be considered within clinical trials (38). Furthermore, different bioactive nanoparticles can deliver drug more precisely, overcome biological barriers, amplify anticancer signaling pathways, remodel the immune microenviroment, and modulate osteogenic catalytic processes, et al. (39).

Preoperative needle biopsy has become a standard clinical practice in the diagnosis of breast tumors, as it allows pathologists to provide essential information on tumor type and biomarker status. However, as also noted in the NCCN guidelines (V5.2024), needle biopsy is not always a definitive diagnostic option. In such cases, excision biopsy serves as an alternative option for accurate pathological classification. According to the same NCCN guidelines, radical surgery, total axillary lymph node dissection, and adjuvant chemotherapy are not routinely recommended for malignant phyllodes tumors. However, our present case and previous literature showed that the clinical behavior varies significantly across different histological subtypes. In aggressive subtype, such as heterologous osteosarmotous differentiation, more extensive surgical resection and aggressive systemic therapy should be administered. Preoperative imaging, particularly DCE-MRI could effectively evaluate the vascular pattern masked by the diffuse calcification, which portends the rapid growth and aggressive biological behavior. Postoperative histopathological examination is of importance to identify the high-risk subtypes. Finally, communication between the oncologist and the patient about the prognosis and individualized treatment strategies is crucial for optimal clinical management.

## Conclusion

MPT with heterologous osteosarcomatous differentiation is a rare disease entity with a dismal prognosis, even when managed aggressively. Unlike other reported cases, the present case was characterized by heterologous osteosarcomatous components constituting nearly the entire tumor volume; moreover, its pathological basis-particularly the rich vascularity- was detected only through DCE-MRI. Intrathoracic metastases exhibited imaging features similar to the primary tumor. We should integrate various information of comprehensive imaging and meticulous pathological examination to make a definitive diagnosis. Aggressive treatment and vigilant monitoring measures are essential to improve outcomes in this rare disease.

## Data Availability

The original contributions presented in the study are included in the article/supplementary material. Further inquiries can be directed to the corresponding author.
